# Occurrence dataset of protected fungal species for the Red Data Book in Yugra Region, Western Siberia

**DOI:** 10.3897/BDJ.13.e155657

**Published:** 2025-07-10

**Authors:** Nina V. Filippova, Elena A. Zvyagina, Sergey Yu. Bolshakov, Stanislav P. Arefyev, Tatiana M. Bulyonkova, Ilya V. Filippov, Yury A. Rebriev, Anton G. Shiryaev, Iraida V. Stavishenko, Tatyana Yu. Svetasheva

**Affiliations:** 1 Yugra State University, Khanty-Mansiysk, Russia Yugra State University Khanty-Mansiysk Russia; 2 Moscow State University, Moscow, Russia Moscow State University Moscow Russia; 3 Komarov Botanical Institute of the Russian Academy of Sciences, Saint Petersburg, Russia Komarov Botanical Institute of the Russian Academy of Sciences Saint Petersburg Russia; 4 Institute of the problems of northern development, Tyumen, Russia Institute of the problems of northern development Tyumen Russia; 5 A.P. Ershov Institute of Informatics Systems Russian Academy of Sciences, Novosibirsk, Russia A.P. Ershov Institute of Informatics Systems Russian Academy of Sciences Novosibirsk Russia; 6 Southern Scientific Centre of Russian Academy of Sciences, Rostov-on-Don, Russia Southern Scientific Centre of Russian Academy of Sciences Rostov-on-Don Russia; 7 Institute of Plant and Animal Ecology of Ural branch of RAS, Ekaterinburg, Russia Institute of Plant and Animal Ecology of Ural branch of RAS Ekaterinburg Russia; 8 Tula State Lev Tolstoy Pedagogical University, Tula, Russia Tula State Lev Tolstoy Pedagogical University Tula Russia

**Keywords:** GBIF, fungal conservation, macrofungi, mushrooms, protected species, open biodiversity data, biodiversity informatics

## Abstract

**Background:**

The data paper describes a dataset of occurrences of fungal species listed in the Red Data Book of Yugra Region (Western Siberia, Russia). The dataset is based on all digitised records of fungal occurrences in the region. The authors conducted an assessment of the conservation status of fungal species for the revised third edition of the Red Data Book of Yugra. The third edition of the Red Data Book of Yugra includes a total of 61 fungal species (excluding lichens). Of these, nine species are listed on the IUCN Red List and six are included in the Red Data Book of Russia. At the time of publication, the dataset comprises 1180 records of protected species, including human observations, preserved specimens and material citations from literature.

**New information:**

The paper provides the first overview of the history of fungal conservation in Yugra (Khanty-Mansi Autonomous Okrug–Yugra, KhMAO-Yugra). For the first time, open-source data are used for the assessment of the occurrence of rare species and evaluation of their conservation status for the revised third edition of the Red Data Book of Yugra. An integrated occurrence dataset for the species included in the new edition of the Red Data Book is presented.

## Introduction

Conservation efforts focused on fungi started significantly later than those for animals and plants (Moore et al. 2001). Since most fungi exist as microorganisms, along with their vast diversity and limited study in many regions, many challenges arise in implementing conservation initiatives in this group. However, several studies documented the disappearance of some species of fleshy fungi (macrofungi, macromycetes) (Kirk et al. 2008) from their habitats around the beginning of the 21^st^ century (Arnolds 1991). The most common reason for extinction is habitat loss or degradation for habitat-specific species. Many groups of fungi have narrow ecological niches and are linked to habitats that quickly disappear due to land use, such as old-growth forests, nutrient-poor soils, old pastures or peatlands (Arnolds 2001, Moore et al. 2001, Dahlberg et al. 2010, Dahlberg and Mueller 2011).

Conservation efforts and the establishment of Red Lists globally depend on the documentation of changes in population size and global distribution area of protected species. However, evaluating the population status of fungi comes with several challenges, as reported by different authors (Moore et al. 2001, Heilmann‐Clausen et al. 2014, May et al. 2018):

1. Fungi are modular organisms, with individual size being unstable and varying significantly from group to group;
2. Fungi have broad geographic ranges and the coverage of spore dispersal can be extensive;
3. Cryptic lifestyle makes it challenging to reliably record the presence of the organism in nature (the absence of registration does not imply that the species is not living hiddenly in the environment);
4. Fungal systematics is undergoing major changes and shifts due to the fast progress of molecular phylogeny methods;
5. Many regions remain under-explored, fungal diversity-wise;
6. Ecological groups of fungi are highly diverse, including a wide range of phytopathogens, microscopic soil fungi and other groups for which traditional conservation measures are not applicable.

Nevertheless, fungal conservation has developed various approaches to assess the conservation status in some fungal groups. A unified approach to population assessment has been employed (Dahlberg and Mueller 2011). Extensive effort has been made to document changes in the abundance of various species over the last decades in Europe and America (Kotiranta 2001, Molina 2008, Dahlberg et al. 2010, Tiezheng et al. 2020). Online databases have been established for recording findings of rare fungal species (Lofgren and Stajich 2021). In most countries, fungi are presently included in regional and statewide Red Lists alongside plants and animals. The Fungal Conservation Committee of the IUCN Species Survival Commission began work on the evaluation of the global conservation status of fungi (Mueller et al. 2022). Currently, there are 1300 fungal species assessed and published (IUCN 2025).

### Fungal conservation in Russia and Khanty-Mansi Autonomous Okrug-Yugra Region

The first Red Data Book of fungi of the Russian Federation was published in 2008 and included 66 species (42 species are lichens) (Bardunov and Novikov 2008), the recently published second edition featuring 115 species (including 74 lichens) (Geltman 2024). The majority of regional Red Data Books in Russia have chapters dedicated to fungi (Svetasheva 2013). The national database of regional Red Lists of fungi (Bolshakov S.Yu., unpublished materials) includes approximately 2200 species. Of this number, fleshy fungi (excluding lichens) constitute about 1200 species, which is over a third of the entire diversity of fleshy fungi in Russia (Bolshakov et al. 2021). The numbers of species of fungi in the regional Red Data Books vary from 4 - 247 (on average, 68 species amongst 84 regional Red Data Books).

Mycological research in Western Siberia has a relatively short history. The first edition of the Red Data Book of Yugra (Vasin 2003) included 24 species of fungi (without lichens), presented in detail and separated into different groups in Table 1.The second edition of the Red Data Book of Yugra included 54 species (without lichens) (Vasin and Vasina 2013); the species composition changed significantly with 43 new and excluded 14 species. In accordance with the federal legislation, the regional Red Data Book is re-issued once every ten years. The next, most recent edition covers 61 species (Tables 1, 2), including 21 species in the monitoring list (these species are not formally protected, but are listed as candidates for inclusion in the List and published in the appendix to the Red Data Book).

### Open biodiversity data in the northern part of Western Siberia

Advances in biodiversity informatics methods and the introduction of key principles of FAIR data in the last decade have produced powerful new instruments for species conservation. Open data serve for assessing population dynamics, verifying species occurrences over time, supporting global models and delineating species ranges and are used as a source of information for timely decision-making. Mobilisation of biodiversity data in the region of Western Siberia began quite recently (Filippova et al. 2020a). This includes the current active effort for digitising data of different types of fungal occurrences:

1. Major regional collections of fungi were logged into databases and made publicly accessible through the Global Biodiversity Information Facility platform. In total, these collections amount to approximately 12 thousand specimens (Filippova et al. 2022a).
2. Over the course of several years, literature-based occurrence data for the region have been digitised; information from about 100 published sources is presented as a dataset and available through GBIF (approximately 5 thousand occurrences) (Filippova et al. 2020).
3. Citizen-science observations of fungi on the iNaturalist.org platform have been integrated and verified and the data published as a dataset: it includes about 13 thousand finds, with about 200 finds of species from regional, national or IUCN Red Lists (Filippova et al. 2022).
4. As a result of these data mobilisation initiatives, many individual research projects adopted the protocols of publishing data from fungal inventories and monitoring as open-access datasets through GBIF. About 10 thousand finds can be attributed to such individual projects.

There are now approximately 63K occurrences openly available through GBIF regarding finds of protected fungal species in Yugra. We believe that these data should be used in conservation efforts, particularly for regional Red List assessments.

## Sampling methods

### Study extent

The objective of this initiative was to integrate open data on occurrences of protected fungal species in the region, utilise these data to assess the conservation status of species for the third edition and generate a dataset encompassing all occurrences of species listed in the current edition. Such a dataset can be treated as an electronic appendix containing the original occurrence records for the Red Data Book publication.

### Step description

**Figure 1.**
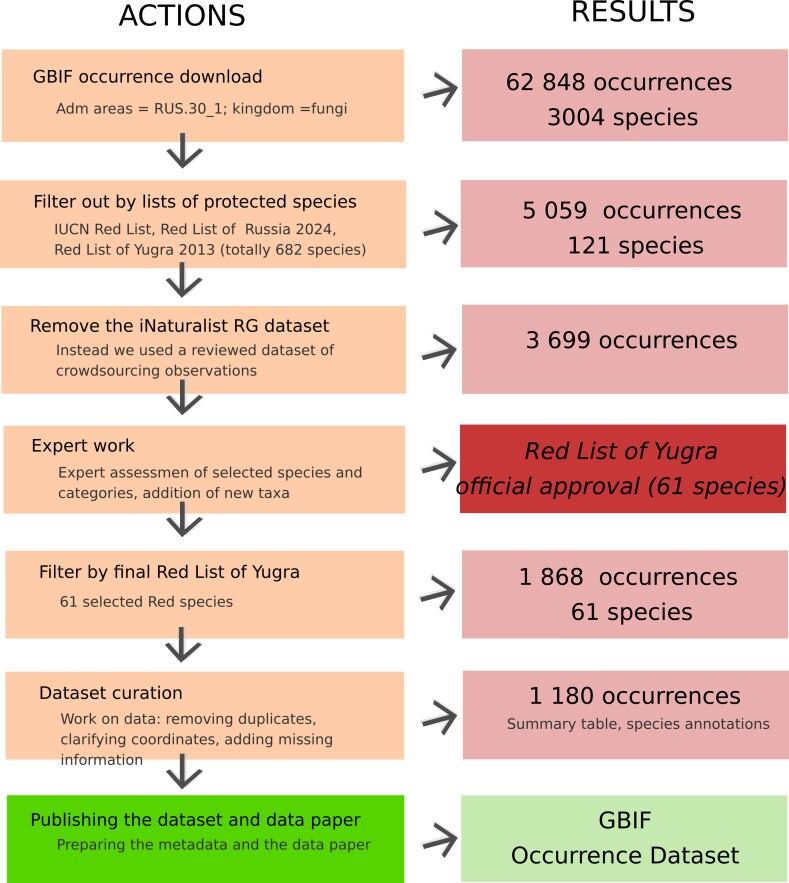
Actions and results of preparing data on the occurrences of fungal species included in the third edition of the Red Data Book of Yugra.

1. Data available through GBIF were downloaded, reviewed, cleaned and used for the assessment of species conservation status (Fig. 1). The data download from GBIF used geography and taxonomy filters to create **a working dataset** (*administrativeAreas = “Khanty-Mansi Autonomous Okrug”, kingdom = “fungi”, taxonRank = “species*”). All datasets were included in the download, except iNaturalist research grade observations (iNaturalist 2024). Nonetheless, observations from iNaturalist were presented in the revised iNaturalist-based observation dataset previously published through GBIF Filippova et al. 2022b). The download fields included the following: *gbifID, scientificName, kingdom, class, order, family, genus, specificEpithet, decimalLatitude, decimalLongitude, bibliographicCitation, datasetName, coordinateUncertaintyInMeters, locality, habitat, eventDate.* The primary occurrence download could be accessed at (GBIF.org 2023). A dozen new occurrences of protected species absent in GBIF were later added from unpublished sources (reports by the authors of the present publication).
2. **The reference dataset** of published Red Lists of fungi was created, including: 1) the IUCN Red List (IUCN 2024); 2) the Red Data book of the Russian Federation (Geltman 2024); and 3) the Red Data Book of Khanty-Mansi Autonomous Okrug, second edition (Vasin and Vasina 2013). All taxonomic names were synonymised using the GBIF species matching tool; the resulting reference dataset included 943 records (Suppl. material 1).
3. **The working dataset** was filtered by taxa presented in the reference dataset and amended by two fields (Red Data Book name and protection category). Additional rare indicator species not covered by any of the Red Lists were picked, based on opinions of several experts on the fungal diversity of the region.
4. For the purpose of standardisation, we created additional fields with calculated coordinates after clustering the original occurrence coordinates. The analysis based on Python’s scikit-learn library (Pedregosa et al. 2011), specifically using DBSCAN from sklearn.cluster for clustering and haversine_distances from sklearn.metrics.pairwise to compute geodesic distances between geographic coordinates. The dataset was amended by four fields: 5 km cluster ID, 5 km cluster coordinates, 25 km cluster ID, 25 km cluster coordinates. These data were used to calculate the number of localities (we defined a locality as a 5 km radius area) and to plot points on the map in the Red Data Book (represented as a 25 km radius at the map’s scale).
5. The evaluation of conservation status was performed according to the following protocol. The IUCN criteria (Dahlberg and Mueller 2011) is hardly applicable in this region, as it remains largely undeveloped. However, when making the assessment, the following criteria were used: 1) the species must be large (macromycetes) and sufficiently recognisable; 2) small number of registered localities in the region; 3) the species serves as an indicator of valuable, rare substrate types and habitats; and 4) the species may be threatened by commercial harvesting. Species listed in the national Red Data Book were almost always included in the regional list. If a species is included in the IUCN Red List, this may also warrant its inclusion (provided other parameters are met).
6. A summary table was compiled, including the following information: a) number of localities for each taxon; b) districts of occurrence for each taxon; c) presence of the taxon within boundaries of protected natural areas; d) nationwide distribution of each taxon according to Bolshakov et al. (2021); and e) presence of the taxon in the national Red Data Book, IUCN Red List and regional Red Data Books of the adjacent regions (Suppl. material 2).
7. **The working dataset** was checked, data cleaning was applied and **the final dataset** was published through GBIF (Filippova et al. 2025c).

## Geographic coverage

### Description

**Figure 2.**
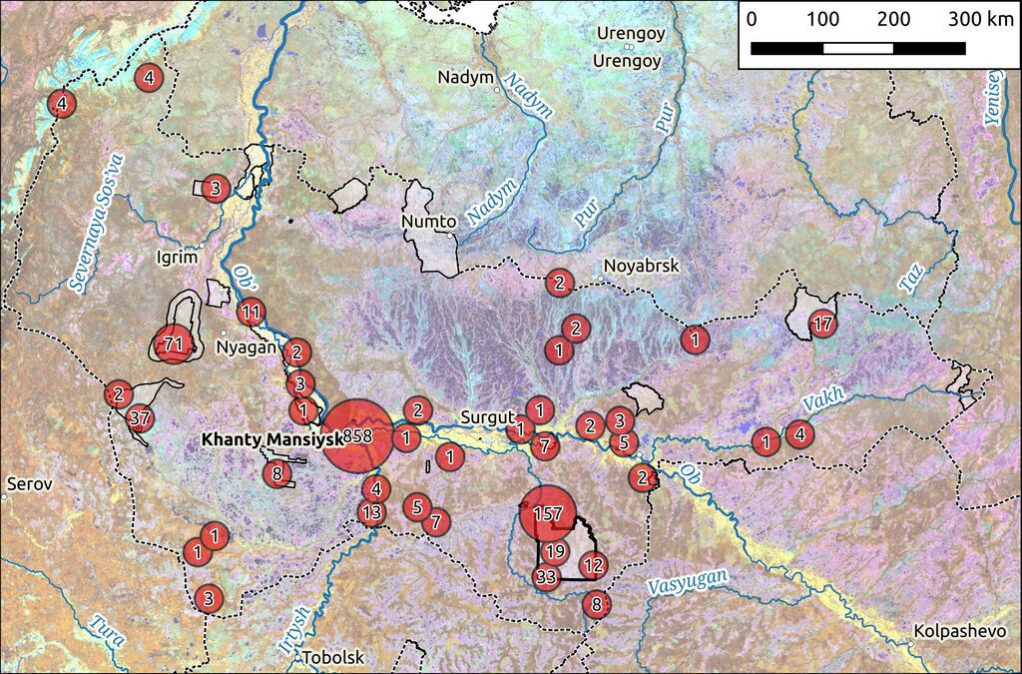
The distribution map of the resulting occurrence dataset of protected fungal species of Yugra on MODIS satellite image with the boundaries of Nature Protected areas also displayed.

The geographic distribution of the Red-Listed fungal species of Yugra in the resulting occurrence dataset covers all nine administrative districts of Khanty-Mansi Autonomous Okrug. The number of findings is unevenly distributed, reflecting varying research activity in different parts of the region (Fig. 2). Approximately a half of all occurrences were made in the Khanty-Mansiyskiy District, 17% in Surgutskiy and 12% in Sovetskiy District. The remaining six districts account for about 8% of all records (Table 3). The data exhibit significant bias towards one particular district. This implies that the distribution patterns may change if other districts are more thoroughly investigated in future studies. On the other hand, for more rigorous research on rare species distributions, habitat modelling could be employed to predict their occurrence in understudied areas.

### Coordinates

57.4 and 65.14 Latitude; 59.6 and 81.7 Longitude.

## Taxonomic coverage

### Description

The taxonomic structure of the third edition of the regional Red Data Book comprises 61 species of fungi (excluding lichens). They belong to two phyla: Ascomycota (8 species) and Basidiomycota (54); 14 orders (the most represented are the Agaricales and Polyporales, with 19 species in each); 35 families (Polyporaceae – 8 species, Hygrophoraceae – 6 species and others); 51 genera (3 genera represented by 3 species, 5 genera – by 2 species and the rest represented by a single species).

## Usage licence

### Usage licence

Creative Commons Public Domain Waiver (CC-Zero)

## Data resources

### Data package title

GBIF occurrence dataset

### Resource link

https://doi.org/10.15468/4c7z66

### Alternative identifiers

https://www.gbif.org/dataset/613eb74b-d32a-494a-b5d8-9a952035038c; http://ipt.ugrasu.ru:8080/manage/resource.do?r=redbookyugra

### Number of data sets

1

### Data set 1.

#### Data set name

Occurrence dataset of protected fungal species for the Red Data Book in Yugra Region, Western Siberia

#### Data format

Darwin Core

#### Character set

UTF-8

#### Download URL

http://ipt.ugrasu.ru:8080/archive.do?r=redbookyugra

#### Description

The dataset includes 21 fields and 1180 records on findings of fungal species listed in the third edition of the Red Data Book of Yugra. The core information about an occurrence covers the scientific name, bibliographic reference (if published in scientific literature), database references (if published in a dataset), geographical location of the find, date, presence of specimens in collections and authorship.

**Data set 1.** 

Column label	Column description
occurrenceID	A unique identifier for the occurrence.
ScientificName	The full scientific name.
taxonRank	The taxon rank.
kingdom	The taxon kingdom (fungi).
collectionCode	The collection code for voucher specimens (five collection codes listed in the dataset).
basisOfRecord	The nature of the record (material citation, human observation, preserved specimen, material sample).
associatedReferences	A related resource where the occurrence was cited (GBIF datasets).
bibliographicCitation	A literature reference if the occurrence has been published (for material citation records).
catalogNumber	The collection catalogue number.
recordedBy	A person who recorded the occurrence.
institutionCode	The name of the institution in which the collection specimens are stored.
stateProvince	The name of administrative region (Region).
locality	The locality name or topographic description, in English.
decimalLatitude	The geographic latitude.
decimalLongitude	The geographic longitude.
coordinateUncertaintyInMetres	Coordinate uncertainty.
geodeticDatum	The geodetic datum of the coordinate.
countryCode	The standard code for the country (RU).
eventDate	The date during which a collection occurred.
dateIdentified	Date when the occurrence was identified.
identifiedBy	A person who identified the occurrence.

## Additional information

### Results

The mobilisation of biodiversity data for Yugra enabled unified access to approximately 63 thousand occurrences openly available in GBIF. This information was used to assess the list of protected fungi for the third edition of the regional Red List. About 13% of observations come from iNaturalist.org research grade observations and were excluded from the revision at first (except iNaturalist-based occurrences which were reviewed earlier in a dedicated project (Filippova et al. 2022b)). The contribution of individual datasets to the total number of occurrences is shown in Table 4. Of them, 42% were represented by observations, mainly on iNaturalist.org or in plot-based monitoring counts (Human observation), 37% by literature-based reports (Material citation) and 21% by preserved collection specimens (Preserved specimen).

After filtering down to the species level, about 46K records were selected for further analysis. These were further filtered to include only species from the IUCN Red List, the Red Data Book of Russia, the previous edition of the regional Red Data Book and selected high-value habitat indicator species, which resulted in a set of about 1.8K occurrences.

This dataset was used to assess the protection status and create the species annotations. Following a check and final filtration of the dataset, 1180 records were retrieved and published as a new dataset representing an electronic appendix to the third edition of the Red Data Book of Yugra (section Fungi).

**Figure 4.**
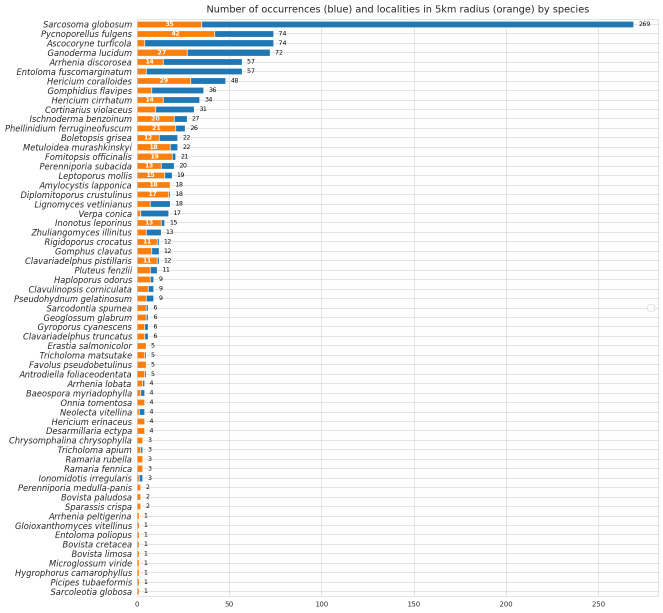
Number of occurrences and calculated localities (in 5 km cluster) by species, included in the third edition of the Red Data Book of Yugra as a result of data mobilisation.

As a result of integrating occurrence data on protected species, the number of records in the third edition of the regional Red Rist of fungi increased threefold compared to the previous edition (Table 5, Fig. 4).

The total number of species in the third edition increased by 17%, from 53 to 61. Six new species were added to the monitoring list, two new species received protection status III and one protection status IV. The protection status of one species (*Sarcosomaglobosum*) was downgraded from III to IV due to the high number of newly-registered localities. For two species, the status was upgraded from IV to III (*Ionomidotisirregularis*, *Baeosporamyriadophyla*). One species in the third edition (*Arrheniapeltigerina*) was moved from the status III to the monitoring list due to insufficient distribution data.

New species on the list were added based on their status in the IUCN Red List or the national Red Data Book:

- category III – two species included in the IUCN Red List; rare and treated as high-value habitat indicators (*Bovistapaludosa, Tricholomaapium*);
- category IV – one species included in the IUCN Red List and the Federal Red Data Book; treated as vulnerable due to the possibility of commercial overharvesting (*Tricholomamatsutake*);
- monitoring list – considered rare and viewed by experts as indicators of specific high-value habitats. Three species were included in the IUCN Red List (*Gloioxanthomycesvitellinus*, *Hygrophoruscamarophyllus*, *Perenniporiamedulla-panis*); others are not protected by any list, but considered rare in the region (*Microglossumviride*, *Bovistacretacea*, *Lignomycesvetlinianus*).

The regional list of protected species includes nine species listed in the IUCN Red List and six fungal species included in the national Red Data Book (Table 6).

The ecology and habitat specificity of the species included in the Red Data Book of Yugra are quite diverse. About 70% of the species listed are saprotrophs (mainly wood decomposers), 20% form mycorrhizal associations with different trees and a few species are parasites on living trees. About a half of all protected species inhabit old-growth (coniferous) forests and 10% grow in peatlands.

Speaking of potential threats to the Red-Listed species, most are considered vulnerable because of habitat loss worldwide (logging, peat excavation, loss of large woody debris), while three of the species are treated as threatened due to commercial harvesting of fruitbodies worldwide and in the region. Most of the species have been recorded in one or several protected natural areas (Table 7), which contributes to the conservation of their populations and habitats.

## Supplementary Material

B728A8E7-2D47-571F-97A6-40354D2B9427 10.3897/BDJ.13.e155657.suppl1 Supplementary material 1 Reference table of fungal species included in the regional, national and IUCN Red Lists and their protection status Data type dataset Brief description The table includes lists of species and their protection status from four Red Lists (IUCN, Red List of Russian Federation, Red List of Yugra (2013), Red list of Yugra (2024)). The table presents a total of eight fields and 943 records. The fields include: Red List name, year of publication, protection status, protection category, scientific name and fungal group. File: oo_1304850.csv https://binary.pensoft.net/file/1304850 Bolshakov S.Yu., Filippova N.V.

67E41DB2-F57C-531A-B3E6-521E90410231 10.3897/BDJ.13.e155657.suppl2 Supplementary material 2 Summary table Data type dataset Brief description The table includes summary information about species listed in the third edition of the Red List of Yugra, comprising a total of 13 fields and 61 records. The fields include: scientific name, vernacular name, number of occurrences, number of unique localities (5 km clustering), number of unique counties, list of counties, protection status in the Red List Yugra, IUCN status (if available), ecological group, habitat and potential threats. File: oo_1351844.csv https://binary.pensoft.net/file/1351844 Filippova N.V., Bolshakov S.Yu.

## Figures and Tables

**Figure 3.**
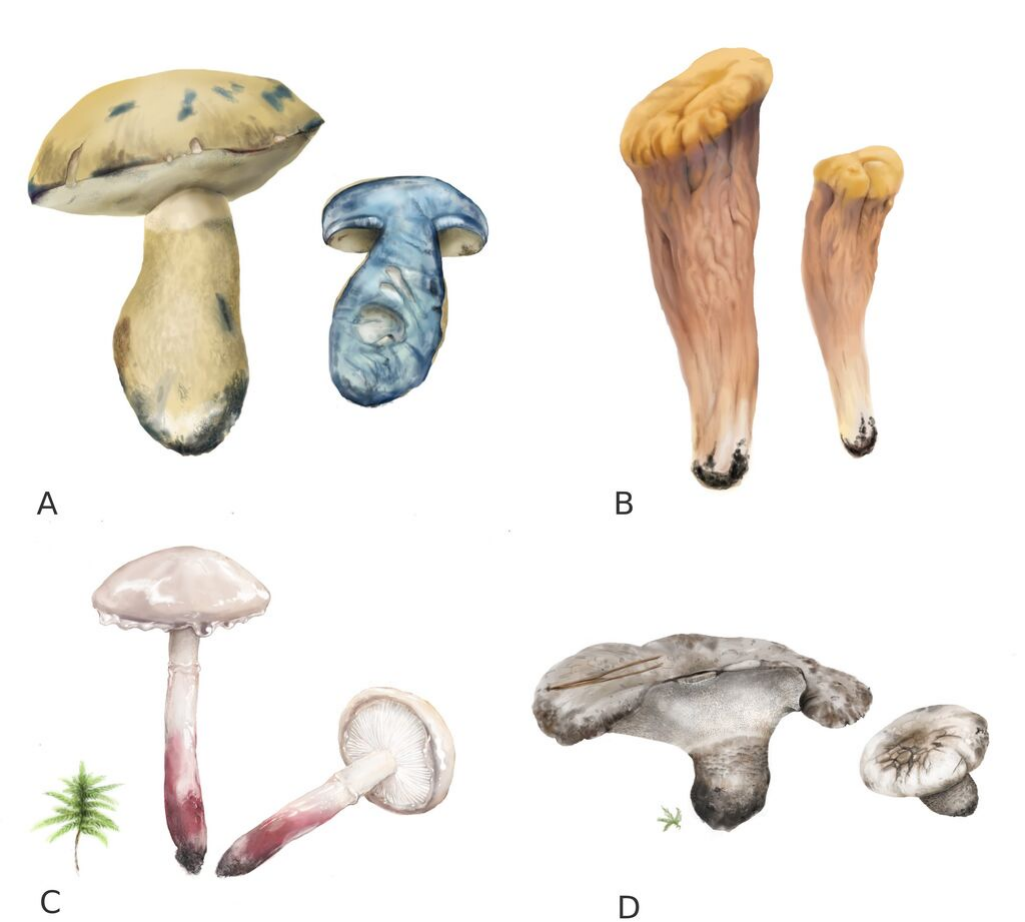
Illustrations of selected fungal species included in the third edition of the Red Data Book of Yugra (drawings by T. Bulyonkovа): **A** *Gyroporuscyanescens*; **B** *Clavariadelphuspistillaris*; **C** Zhuliangomycesillinitusvar.rubescens; **D** *Boletopsisgrisea*.

**Table 1.** The number of fungal species by groups in three editions of the regional Red Data Books of Yugra.

**Regional Red Data Books editions**	Vasin (2003)	Vasin and Vasina (2013)	Starikov (2024)
**Total number of species**	32 + 9 in monitoring list	67 + 15 in monitoring list	40 + 21 in monitoring list
**Aphyllophoroid**	22	30	32
**Agaricoid**	1	15	17
**Ascomycetes**	1	7	8
**Lichens** (excluded from analyses in present publication)	15	28	18
**Other groups**	0	2	4

**Table 2.** The number of fungal species (without lichens) by protection status in three editions of the regional Red Data Book of Yugra.

**Protection status**	Vasin (2003)	Vasin and Vasina (2013)	Starikov (2024)
**II**	2	2	2
**III**	30	32	30
**IV**		5	8
**Monitoring list**	9	15	21
**Totally**	41	54	61

**Table 3.** Number of occurrences of protected species by administrative districts of the Khanty-Mansi Autonomous Okrug-Yugra.

№	**District**	**Number of occurrences**	%
1	Khanty-Mansiyskiy District	748	63
2	Surgutskiy District	199	17
3	Sovetskiy District	139	12
4	Nizhnevartovskiy District	57	5
5	Berezovskiy District	21	2
6	Oktyabrskiy District	10	0.8
7	Nefteyuganskiy District	9	0.8
8	Kondinskiy District	2	0.2
9	Nizhnetavdinskiy District	1	0.1
10	Beloyarskiy District	1	0.1

**Table 4.** Filippova et al. (2024c)Contribution of individual datasets published through GBIF to working dataset to assess protection status of fungi for new edition of Red Data Book in Yugra.

**Dataset name**	**Number of observations**	**Reference**
Fungal literature records database of the northern West Siberia (Russia)	825	Filippova et al. (2025d)
Crowd-sourcing fungal biodiversity: revision of iNaturalist observations in north-western Siberia	372	Filippova et al. (2024c)
The Fungarium of Yugra State University	165	Filippova et al. (2025a)
Plot-based observations of macrofungi in raised bogs in Western Siberia (2014-2024)	64	Filippova et al. (2025b)
Plot-based observations of terrestrial macrofungi in different forest types of boreal zone in West Siberia (2015-2024)	61	Filippova et al. (2024b)
The fungarium of Kondinskie Lakes Natural Park	10	Butunina (2025)
The Fungarium of Elena Zvyagina	4	Zvyagina (2025)
The Bovista species diversity in Russia, based on the data of official herbaria (LE, VLA, KEM, MAG, NSK, K, PRM) and the author's personal mycological collection (YuR)	4	Rebriev (2024)
The checklist of macrofungi of raised bogs: barcoding of accumulated collection following the 9-year plot-based monitoring in north-western Siberia	3	Filippova et al. (2024a)
Collection and observation of macrofungi from Vakh River Basin (Western Siberia)	2	Zvyagina (2024)
INSDC Sequences	1	European Bioinformatics Institute (EMBL-EBI) and GBIF Helpdesk (2025)
Field workshop on data publishing in Ethnographic and ecological park "Yugra" (Megion, 1-3 July 2019). Biodiversity assessement	1	Mingalimova et al. (2024)

**Table 5.** Lists of fungi (without lichens) and numbers of occurrences in three editions of the regional Red Data Book of Yugra.

**Species**	Vasin (2003)	Vasin and Vasina (2013)	Starikov (2024)
*Abortiporusbiennis* (Bull.) Singer	1		
*Amylocystislapponica* (Romell) Bondartsev & Singer	6	18	18
*Antrodiellafoliaceodentata* (Nikol.) Gilb. & Ryvarden		2	5
*Arrheniadiscorosea* (Pilát) Herink & Kotl.		9	57
*Arrhenialobata* (Pers.) Kühner & Lamoure ex Redhead		2	4
*Arrheniapeltigerina* (Peck) Redhead, Lutzoni, Moncalvo & Vilgalys		1	1
*Ascocoryneturficola* (Boud.) Korf		5	74
*Baeosporamyriadophylla* (Peck) Singer		1	4
*Boletopsisgrisea* (Peck) Bondartsev & Singer (Fig. 3)		1	22
*Bovistacretacea* T.C.E.Fr.			1
*Bovistalimosa* Rostr.		1	1
*Bovistapaludosa* Lév.			2
*Chrysomphalinachrysophylla* (Fr.) Clémençon		3	3
*Clavariadelphuspistillaris* (L.) Donk		14	12
*Clavariadelphustruncatus* Donk (Fig. 3)		1	6
*Clavulinopsiscorniculata* (Schaeff.) Corner		4	9
*Climacodonseptentrionalis* (Fr.) P. Karst.	2		
*Cortinariusviolaceus* (L.) Gray	1	7	31
*Desarmillariaectypa* (Fr.) Lamoure		1	4
*Diplomitoporuscrustulinus* (Bres.) Domański		18	19
*Entolomafuscomarginatum* P.D.Orton		3	58
*Entolomapoliopus* (Romagn.) Noordel.		1	1
*Erastiasalmonicolor* (Berk. & M.A.Curtis) Niemelä & Kinnunen		9	8
*Favoluspseudobetulinus* (Murashk. ex Pilát) A.B.De		2	5
*Fomitopsisofficinalis* (Vill.) Bondartsev & Singer	6	10	39
*Ganodermalucidum* (Curtis) P.Karst.	5	17	75
*Geoglossumglabrum* Ehrenb.		1	6
*Gloioxanthomycesvitellinus* (Fr.) Lodge, Vizzini, Ercole & Boertm.			1
*Gomphidiusflavipes* Peck		5	36
*Gomphusclavatus* (Pers.) Gray		2	12
*Gyroporuscyanescens* (Bull.) Quél. (Fig. 3)		2	6
*Haploporusodorus* (Sommerf.) Bondartsev & Singer		5	9
*Hericiumcirrhatum* (Pers.) Nikol.		9	34
*Hericiumcoralloides* (Scop.) Pers.	8	22	50
*Hericiumerinaceus* (Bull.) Pers.		3	4
*Hygrophoruscamarophyllus* (Alb. & Schwein.) Dumée, Grandjean & Maire			1
*Inonotusleporinus* (Fr.) Gilb. & Ryvarden		11	15
*Ionomidotisirregularis* (Schwein.) E.J.Durand			3
*Ischnodermaresinosum* (Schrad.) P. Karst.	7		
*Ischnodermabenzoinum* (Wahlenb.) P.Karst.		19	29
*Laetiporussulphureus* (Bull.) Murrill	5		
*Leccinumpercandidum* (Vassilkov) Watling	3		
*Leptoporusmollis* (Pers.) Quél.	7	22	20
*Lignomycesvetlinianus* (Domański) R.H.Petersen & Zmitr.		4	18
*Metuloideamurashkinskyi* (Burt) Miettinen & Spirin		24	22
*Microglossumviride* (Pers.) Gillet			1
*Neolectavitellina* (Bres.) Korf & J.K.Rogers		1	4
*Onniatomentosa* (Fr.) P.Karst.		2	4
*Oxyporuspopulinus* (Schumach.) Donk	2		
*Perenniporiamedulla-panis* (Jacq.) Donk			2
*Perenniporiasubacida* (Peck) Donk		20	20
*Phaeolusschweinitzii* (Fr.) Pat.	4		
*Phellinidiumferrugineofuscum* (P.Karst.) Fiasson & Niemelä		23	26
*Polyporustubaeformis* (P.Karst.) Ryvarden & Gilb.		2	1
*Pluteusfenzlii* (Schulzer) Corriol & P.-A.Moreau		7	11
*Pseudohydnumgelatinosum* (Scop.) P.Karst.		2	9
*Pycnoporellusfulgens* (Fr.) Donk	13	36	75
*Ramariafennica* (P.Karst.) Ricken		3	3
*Ramariarubella* (Schaeff.) R.H.Petersen		1	3
*Rigidoporuscrocatus* (Pat.) Ryvarden		11	12
*Sarcodontiaspumea* (Sowerby) Spirin		3	6
*Sarcoleotiaglobosa* (Sommerf.) Korf		1	1
*Sarcosomaglobosum* (Schmidel) Casp.	3	34	289
*Sparassiscrispa* (Wulfen) Fr.		1	2
*Spongipellisspumea* (Sowerby) Pat.	1		
*Tricholomaapium* Jul.Schäff.			3
*Tricholomamatsutake* (S.Ito & S.Imai) Singer			5
*Verpaconica* (O.F.Müll.) Sw.		3	17
*Zhuliangomycesillinitus* (Fr.) Redhead var. rubescens (Fig. 3)		2	13
**Total occurrences by editions**	74	411	1232

**Table 6.** Presence of fungi from regional Red Data Book in higher-status Red Lists.

**Species**	Geltman (2024)	IUCN (2024)
*Sarcosomaglobosum* (Schmidel) Casp.	+	+
*Ascocoryneturficola* (Boud.) Korf	+	
*Desarmillariaectypa* (Fr.) R.A.Koch & Aime	+	
*Ganodermalucidum* (Curtis) P.Karst.	+	
*Gomphidiusflavipes* Peck	+	
*Sparassiscrispa* (Wulfen) Fr.	+	
*Amylocystislapponica* (Romell) Bondartsev & Singer		+
*Arrheniadiscorosea* (Pilát) Zvyagina, A.V.Alexandrova & Bulyonk.		+
*Baeosporamyriadophylla* (Peck) Singer		+
*Boletopsisgrisea* (Peck) Bondartsev & Singer		+
*Fomitopsisofficinalis* (Vill.) Bondartsev & Singer		+
*Haploporusodorus* (Sommerf.) Bondartsev & Singer		+
*Hericiumerinaceus* (Bull.) Pers.		+
*Pluteusfenzlii* (Schulzer) Corriol & P.-A.Moreau		+

**Table 7.** Number of protected species and occurrences registered in protected natural areas in Yugra.

**Nature Protected Area**	**Number of species**	**Number of occurrences**
Nature Park Samarovskiy Chugas	32	188
Nature Reserve Yuganskiy	34	121
Nature Park Kondinskie Ozera	15	45
Nature Reserve Malaya Sosva	22	73
Nature Park Sibirskie Uvaly	16	10
Zakaznik Verkhne-Kondinskiy	5	10
Zakaznik Vaspukholskiy	7	7
Zakaznik Surgutskiy	4	5
Zakaznik Vogulka	5	4
Zakaznik Elizarovskiy	1	1
Nature Monument Ozero Rangetur	1	1
Nature Moniment Un and Ai Novyinklor	1	1
